# Drainage or Packing of the Sella? Transsphenoidal Surgery for Primary Pituitary Abscess: Report of Two Cases

**DOI:** 10.1155/2009/189304

**Published:** 2009-12-16

**Authors:** Soichi Oya, Junichiro Kumai, Taku Shigeno

**Affiliations:** ^1^Department of Neurosurgery, Kanto Rosai Hospital, 211-8510 Kawasaki, Japan; ^2^Department of Neurosurgery, The University of Tokyo, Hongo 7-3-1 Bunkyo-ku, Tokyo 113-8655, Japan

## Abstract

The detailed surgical procedure of the transsphenoidal surgery for pituitary abscess has scarcely been described previously because it is a very rare clinical entity. The authors reported two cases of primary pituitary abscess. In case 1, the anterior wall of the sella turcica was reconstructed with the vomer bone after irrigating the abscess cavity, but the sella was not packed by fat for fear of the persistent infection by devascularized tissues. This led to the postoperative meningocele, the cerebrospinal fluid leak, and bacterial meningitis despite the successful abscess drainage. In case 2, tight sellar packing and reconstruction of the sellar wall were performed to avoid these postoperative complications, which resulted in complete drainage and uneventful postoperative course. Although accumulation of more cases is obviously needed to establish the definitive surgical technique in pituitary abscess surgery, our experience might suggest that packing of the sella is not impeditive for postoperative sufficient drainage.

## 1. Introduction

Treatment for pituitary abscess includes surgical draining of the abscess and antibiotic therapy. The transsphenoidal surgery has been recommended in literature [1, 2], but little has been discussed about the detailed operative procedure. The authors describe two cases of pituitary abscess treated with transsphenoidal surgery with special reference to the sellar packing. 

## 2. Case Reports

### 2.1. Patient 1

**Figure 1:**
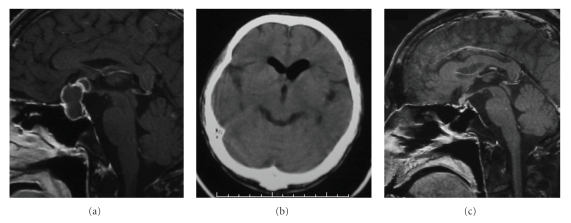
(a) Preoperative sagittal contrast-enhanced T1-weighted MRI demonstrating a ring-enhancing mass in the sella extending to the suprasellar legion. (b) Brain CT scan performed when the CSF rhinorrhea occurred (19 days after the operation) showing pneumocephalus. (c) MRI scan performed at the same time revealing no recurrence of abscess.

A 48-year-old man with a 6-month history of anorexia, lasting mild fever, and general fatigue one month after having his teeth extracted noticed slight impairment of his visual acuity over a month. He had no previous history of immunological disorder including HIV infection. He visited an ophthalmologist and was immediately referred to our hospital because of rapid aggravation of his vision in the preceding few days. The radiological study showed an intrasellar mass with ring-enhancement compressing the optic chiasm on MRI (Figure 1(a)). His visual acuity continued to aggravate very rapidly. He finally became almost blind and comatose on the second day of the admission. He underwent urgent transsphenoidal surgery to diagnose and remove the mass. During surgery, he was diagnosed with a pituitary abscess based on the findings of the white-green pus from the intrasellar cystic mass. The sella was irrigated with copious normal saline after the pus was evacuated. Cerebrospinal fluid (CSF) leakage was not seen intraoperatively. Because there was concern about the recurrence of the abscess, we hesitated using devascularized tissue such as abdominal fat for the intrasellar packing. Therefore, nothing was placed in the sella. The anterior wall of the sella was reconstructed using the vomer bone. Immediately after surgery, the patient's consciousness and visual impairment showed a remarkable improvement. He received intravenous administration of 2 g of cefotiam twice a day for a week. Postoperative MRI showed total drainage of the abscess. No causative agents were identified by Gram-staining or postoperative culture. Nineteen days after the surgery, however, he suddenly presented with CSF rhinorrhea, high fever, and severe headache. The emergency computerized tomography revealed the presence of air in the lateral ventricles (Figure 1(b)). MRI revealed no findings of recurrent abscess (Figure 1(c)). Lumber puncture showed an elevation of cell count to as many as 4250/mm^3^ (99% neutrophils). CSF culture failed to show any bacterial growth. Because these findings indicated severe bacterial meningitis, intravenous antibiotic therapy was immediately initiated using 2 g of penicillin and cefpirome twice a day. His symptoms subsided and the laboratory findings including CSF cell count became normal over a week. He was discharged and has had no recurrence of abscess and meningitis in six months.

### 2.2. Patient 2

**Figure 2:**
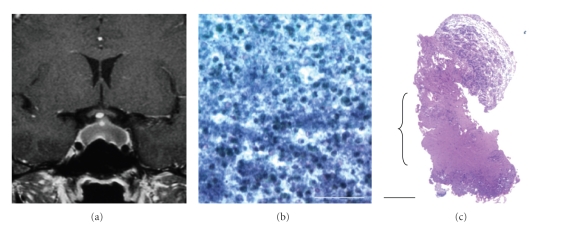
(a) Preoperative coronal contrast-enhanced T1-weighted MRI demonstrating a ring-enhanced mass in the sella. (b) A photomicrograph of aspiration cytology of the pus obtained during the surgery in case 2, demonstrating a cluster of neutrophils: Papanicolaou stain, scale bar; 100 *μ*m. (c) A photomicrograph of a histological cross section from the capsule of the abscess. The capsule of the abscess was the collagen-rich layer (parenthesis). Ciliated columnar epithelium indicating Rathke cleft cyst was not present in any specimens obtained during surgery: H and E staining, scale bar; 200 *μ*m.

A 20-year-old woman with no past medical history visited our hospital presenting with protracted headache, anorexia, paresthesia in upper extremities, and an irregular menstrual cycle over 6 months. She was HIV-negative and had no history of immune deficiency. Her laboratory analysis was normal except for a slight elevation of prolactin (28 ng/mL). A radiological examination revealed that she had a cystic mass in the sella (Figure 2(a)). The ophthalmologic study detected a mild limitation of bitemporal visual field. Because Rathke cleft cyst and cystic pituitary adenoma were the most likely diagnoses, she was observed for 3 months after the examination. Since the headache continued worsening, she agreed to our proposal of the surgical exploration of the sellar mass. A transsphenoidal approach was used and revealed that the mass contained creamy pus. Intraoperative cytological examination showed that the pus contained numerous neutrophils (Figure 2(b)). Based on a tentative diagnosis of pituitary abscess, the sella was irrigated with a large amount of saline and packed tightly with a small lump of abdominal fat this time since we experienced the first case. The anterior wall of the sella was reconstructed using vomer bone, which was sealed by fibrin glue. All her symptoms disappeared immediately after the operation. The cyst wall obtained intraoperatively was collagen (Figure 2(c)), which was consistent with the abscess capsule. There were no findings of ciliated columnar epithelium indicating Rathke cleft cyst. The culture of the pus revealed no infectious agents. She was treated with 1 g of intravenous penicillin and cefpirome twice a day for two weeks after the operation. She experienced no CSF leakage or meningitis with sufficient drainage of the abscess confirmed on postoperative MRI. She was discharged with no neurological deficit and has been in good condition for five months.

## 3. Discussion

Rathke cleft cyst was one of the differential diagnoses in our cases. Although cultures of the pus in both cases were negative, almost half of pituitary abscess cases show no organisms from cultures obtained during surgery [2]. In addition to white-green pus observed during operation, the history of recent tooth extraction and neurological symptoms such as visual loss and disturbed consciousness that extremely rapidly progressed suggested some inflammatory process other than mass effect alone in case 1. In case 2, the microscopic findings of the collagen capsule and neutrophil-rich pus have diagnostic value indicating a pituitary abscess. Microscopic findings of the capsule have been emphasized in the diagnosis of pituitary abscess [3]. The typical ciliated columnar epithelium indicating Rathke cleft cyst was not found in any specimens.

The transsphenoidal approach is recommended since it provides a route for prolonged drainage of the infected agents [4]. Since the pituitary fossa is still an intrinsically infected area even after prompt irrigation, continuous drainage of the remaining abscess is favorable. However, sella packing by the devascularized tissue such as the abdominal fat might render the abscess prone to recur. We therefore hesitated and abandoned packing the sella in case 1, but the case showed severe postoperative bacterial meningitis. The empty sella with inflammatory change might cause descent and twitching of the diaphragm resulting in CSF leak. On the contrary, neither a postoperative CSF leak nor meningitis was seen in case 2 even after packing the sella. The detailed surgical technique has not been discussed in literature because pituitary abscess is a rare clinical entity. Although the necessity of accumulating more cases is obvious, sufficient drainage appears to be achieved in the pituitary abscess surgery even after packing of the sella.

## 4. Conclusion

Based on our experience, packing the sella does not appear to prevent the successful drainage of abscess even in transsphenoidal surgery for pituitary abscess.
